# Association of Genetic Variants in and Promoter Hypermethylation of *CDH1* With Gastric Cancer: A Meta-Analysis

**DOI:** 10.1097/MD.0000000000000107

**Published:** 2014-10-17

**Authors:** Huiquan Jing, Fei Dai, Chuntao Zhao, Juan Yang, Lizhuo Li, Pravina Kota, Lijuan Mao, Kaimin Xiang, Changqing Zheng, Jingyun Yang

**Affiliations:** Institute of Social Science Survey (HJ), Peking University, Beijing; Department of Social Science (HJ), Shenyang Medical College; Emergency Department (LL); Department of Gastroenterology (CZ), Shengjing Hospital, China Medical University, Shenyang, Liaoning; Division of Gastroenterology (FD, JY, LM), Second Affiliated Hospital, Medical College of Xi’an Jiaotong University, Xi’an, Shaanxi; Department of General Surgery (KX), Third Xiangya Hospital, Central South University, Changsha, Hunan, China; Brain Tumor Center (CZ), Cancer and Blood Diseases Institute, Cincinnati Children’s Hospital Medical Center, Cincinnati, OH; Department of Biostatistics and Epidemiology (PK), University of Oklahoma Health Sciences Center, Oklahoma City, OK; Rush Alzheimer’s Disease Center (JYY); and Department of Neurological Sciences (JYY), Rush University Medical Center, Chicago, IL.

## Abstract

Gastric cancer (GC) is a common cause of cancer-related death. The etiology and pathogenesis of GC remain unclear, with genetic and epigenetic factors playing an important role. Previous studies investigated the association of GC with many genetic variants in and promoter hypermethylation of E-cadherin gene (*CDH1*), with conflicting results reported.

To clarify this inconsistency, we conducted updated meta-analyses to assess the association of genetic variants in and the promoter hypermethylation of *CDH1* with GC, including C-160A (rs16260) and other less-studied genetic variants,

Data sources were PubMed, Cochrane Library, Google Scholar, Web of Knowledge, and HuGE, a navigator for human genome epidemiology.

Study eligibility criteria and participant details are as follows: studies were conducted on human subjects; outcomes of interest include GC; report of genotype data of individual genetic variants in (or methylation status of) *CDH1* in participants with and without GC (or providing odds ratios [OR] and their variances).

Study appraisal and synthesis methods included the use of OR as a measure of the association, calculated from random effects models in meta-analyses. We used *I*^2^ for the assessment of between-study heterogeneity, and publication bias was assessed using funnel plot and Egger test.

A total of 33 studies from 30 published articles met the eligibility criteria and were included in our analyses. We found no association between C-160A and GC (OR = 0.88; 95% confidence interval [CI], 0.71–1.08; *P* = 0.215), assuming an additive model (reference allele C). C-160A was associated with cardia (OR = 0.21; 95% CI, 0.11–0.41; *P* = 2.60 × 10^−6^), intestinal (OR = 0.66; 95% CI, 0.49–0.90; *P* = 0.008), and diffuse GC (OR = 0.57; 95% CI, 0.40–0.82; *P* = 0.002). The association of C-160A with noncardia GC is of bottom line significance (OR = 0.65; 95% CI, 0.42–1.01; *P* = 0.054). Multiple other less-studied genetic variants in *CDH1* also exhibited association with GC. Gene-based analysis indicated a significant cumulative association of genetic variants in *CDH1* with GC (all *P*s <10^−5^). Sensitivity analysis excluding studies not meeting Hardy–Weinberg equilibrium (HWE) yielded similar results. Analysis by ethnic groups revealed significant association of C-160A with cardia GC in both Asian and whites, significant association with noncardia GC only in Asians, and no significant association with intestinal GC in both ethnic groups. There was significant association of C160-A with diffuse GC in Asians (*P* = 0.011) but not in whites (*P* = 0.081). However, after excluding studies that violate HWE, this observed association is no longer significant (*P* = 0.126). We observed strong association of promoter hypermethylation of *CDH1* with GC (OR = 12.23; 95% CI, 8.80–17.00; *P* = 1.42 × 10^−50^), suggesting that epigenetic regulation of *CDH1* could play a critical role in the etiology of GC.

Limitations of this study are as follows: we could not adjust for confounding factors; some meta-analyses were based on a small number of studies; sensitivity analysis was limited due to unavailability of data; we could not test publication bias for some meta-analyses due to small number of included studies.

We found no significant association of the widely studied genetic variant C-160A, but identified some other genetic variants showing significant association with GC. Future studies with large sample sizes that control for confounding risk factors and/or intensively interrogate CpG sites in *CDH1* are needed to validate the results found in this study and to explore additional epigenetic loci that affect GC risk.

## INTRODUCTION

Gastric cancer (GC) is one of the most common gastrointestinal malignancies throughout the world. Over the past half century, the incidence of GC has gradually decreased. However, GC remains to be the second most common cause of cancer-related death, with >700,000 deaths/y.^1^ Lauren^2,3^ proposed a histological classification of gastric adenocarcinoma into an intestinal type, including papillary adenocarcinomas and well-differentiated tubular adenocarcinomas, and a diffuse type, including signet ring cell carcinomas and poorly differentiated adenocarcinomas. Based on anatomic conditions, GC can also be divided into 2 subtypes: gastric cardia cancer and noncardia GC, with the former referring to cancers of the top portion of the stomach and the latter referring to cancers in the other areas of the stomach. Noncardia cancer is commonly associated with the *Helicobacter pylori* infection. There was no overall association between gastric cardia cancer and *H pylori* infection, whereas a positive association was observed in high-risk populations.^4^

The etiology and pathophysiology of GC is not fully understood. It is well established that gastric carcinogenesis is a complex multifactorial and multistage process. Previous studies have identified several risk factors that might contribute to gastric carcinogenesis including *H pylori* infection,^5^ inadequate vitamin C uptake,^6^ smoking,^7^ high salt intake,^8^ and low vegetable intake.^9^ Meanwhile, multiple genetic variants and different genetic pathways have been identified to contribute to GC risk,^10^ suggesting that genetic factors play important roles in GC susceptibility. Many studies have been conducted to search for susceptibility genes for GC, such as Interleukin-1, Interleukin-8, Glutathione S-Transferase, and Cytochrome P450 2E1.^11^

E-cadherin glycoprotein, encoded by E-cadherin gene (*CDH1*), is involved in the establishment and maintenance of intercellular adhesion.^12^ In vitro studies found that the A allele of C-160A could decrease the transcriptional efficiency of *CDH1* by approximately 70%, suggesting that the A allele could potentially increase susceptibility to GC.^13^ Many previous studies investigated the association of the genetic variants, C-160A (rs16260) in *CDH1* with GC risk, with conflicting results reported. Several meta-analyses have also been conducted to examine the association of C-160A with GC. Although all of them found no significant association of C-160A with GC, subgroup analysis by ethnic groups reported inconsistent findings (Table 1). In addition to the widely studied genetic variant C-160A, the association between GC and many other less-studied genetic variants in *CHD1* has also been explored in many studies, with inconsistent results reported. Meanwhile, promoter hypermethylation of *CDH1* has also been studied for its effect on GC susceptibility, with inconsistent results found. Therefore, in this study we performed updated meta-analyses to assess the genetic and epigenetic effect of *CDH1* on GC risk. Since GC is a complex disease, a single-nucleotide polymorphism (SNP) may only confer a small or marginal individual effect on GC susceptibility. Studies focused on individual genetic variant may be less powerful in detecting small genetic effect and fail to capture the joint contribution from multiple genetic variants. We therefore conducted a gene-based analysis to examine the cumulative effect of multiple genetic variants in *CDH1* on GC risk.

**TABLE 1:**
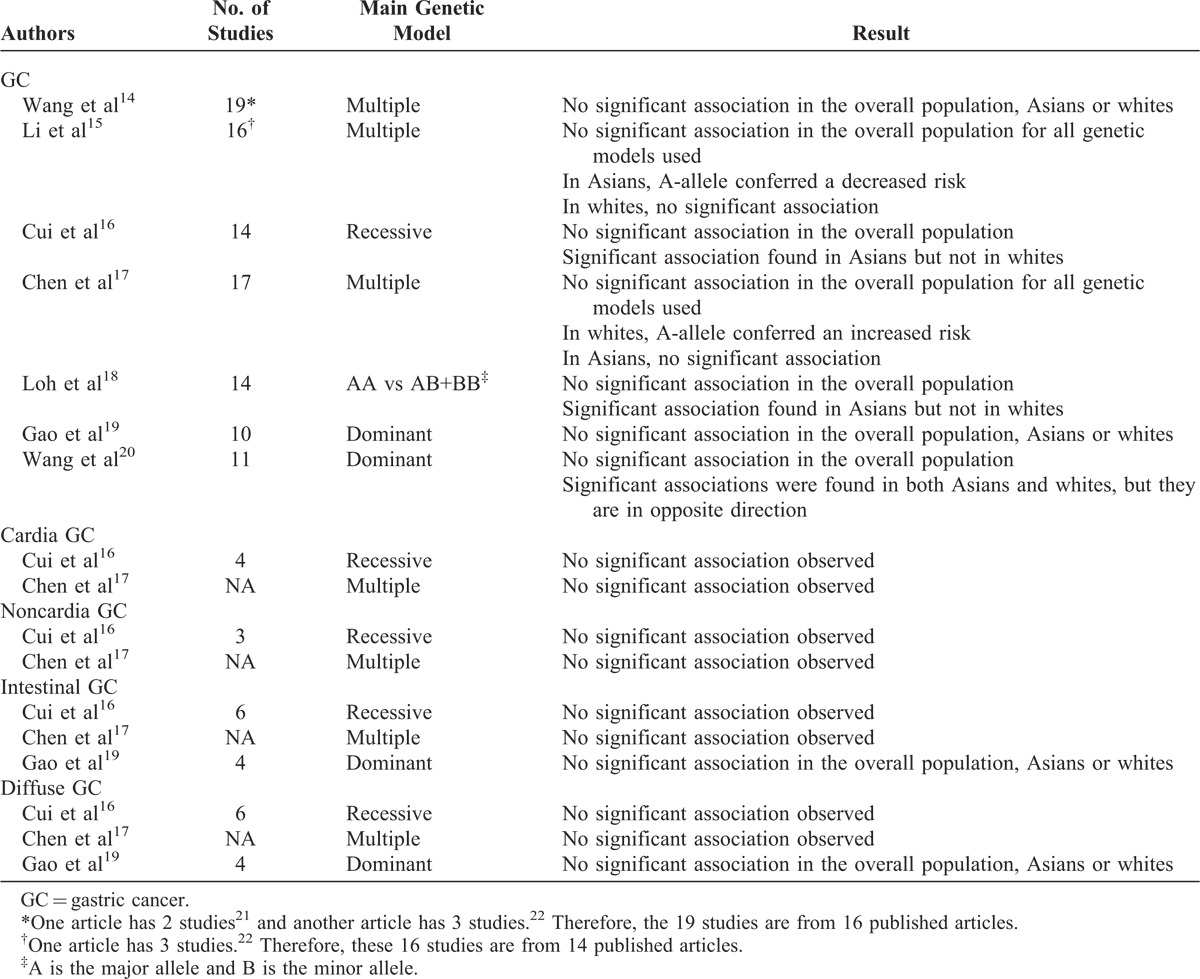
Summary of Previous Meta-Analyses on the Association of *CDH1* C-160A Polymorphism With Risk of GC

## METHODS

### Search Strategy and Study Selection

From January to May 2014, we did an extensive literature search in PubMed, Cochrane Library, Google Scholar, Web of Knowledge, and HuGE, a navigator for human genome epidemiology, for candidate gene studies on the association of GC with genetic variants in and promoter hypermethylation of *CDH1*. Details of keywords used in the literature search can be found in the supplementary file (http://links.lww.com/MD/A54, Key words used in the literature search). We used the following inclusion criteria in determining study eligibility: studies on human subjects, outcomes of interest include GC, and report of genotype data of individual genetic variants in (or methylation status of) *CDH1* in participants with and without GC (or providing odds ratios [ORs] and their variances). All potentially relevant publications were retrieved and further evaluated for inclusion. We also hand-searched references of all relevant publications for additional studies missed by the database search. Only studies published in the English language were included in our analysis. Two authors (H.J. and J.Y.Y.) performed the search independently. Disagreement over eligibility of a study was resolved by discussion until a consensus was reached.

### Data Extraction

Two reviewers (J.Y. and L.M.) independently extracted the following data according to a prespecified protocol: first author’s name, year of publication, characteristics of the study participants (sample size, number of GC patients, and number of participants in the control group, race/country of participants), genotype or methylation status data for subjects with and without GC (or OR and the corresponding variances), and the genetic model used (additive, allelic, dominant, or recessive). Discrepancies were resolved by discussion, and extracted data were entered into a computerized spreadsheet for analysis.

### Statistical Analysis

We used the OR as a measure of the association between the genetic variants in and methylation status of *CDH1* and GC. We used random effects models to calculate ORs and the corresponding 95% confidence intervals (CIs). The inverse of the variance of each study was used as the weight for that study. We used forest plots to graphically represent the calculated pooled ORs and their 95% CIs. Each study was represented by a square in the plot, the area of which is proportional to the weight of the study. The overall effect from the meta-analysis is represented by a diamond, with its width representing the 95% CI for the estimate. We used *I*^2^ for assessment of between-study heterogeneity, and publication bias was assessed using funnel plot and Egger test, and a *P* value <0.20 was considered statistically significant.

We performed an updated meta-analysis for the association of C-160A with GC, and also conducted meta-analysis for association of other genetic variants in *CDH1* with GC, when there are multiple eligible studies for the genetic variants. Otherwise, we compiled the results of the association with GC for genetic variants that appear in single studies. We also analyzed the association between C-160A and subtypes of GC (cardia and noncardia GCs and intestinal and diffuse GCs). Meta-analyses were conducted when there were multiple studies for the analysis of each subtype.

In order to assess the cumulative association of *CDH1* with GC, we conducted a gene-based analysis using the *P* values for the association of individual genetic variants in *CDH1* with GC, calculated from our meta-analyses and/or from published literature. We used 4 popular *P* value combination methods to assess this cumulative association: the Fisher method,^23^ the Simes method,^24^ the modified inverse normal method,^25^ and the truncated product method (TPM).^26,27^ A detailed description of the 4 methods has been reported elsewhere.^26,28^ We used 100,000 simulations to estimate the combined *P* value for TPM because the individual *P* values are most likely to be dependent.

Finally, we performed meta-analysis to examine the effect of promoter hypermethylation of *CDH1* on susceptibility of GC.

### Sensitivity Analysis

We performed separate meta-analyses by excluding studies in which genotype in the control group did not meet Hardy–Weinberg equilibrium (HWE). We also performed meta-analysis separately for individual ethnic groups/countries of origin (Asian and whites).

As a research using systematic review and meta-analysis, ethical approval of this study is not required. This work was reported according to the PRISMA guidelines.^29^ Meta-analysis was performed using Stata 11.2 (StataCorp LP, College Station, TX). All other analyses were performed using SAS version 9.3 (SAS Institute Inc, Cary, NC), R (www.R-project.org), and Matlab 8.1.0.604 (The MathWorks, Inc, Natick, MA). A *P* value <0.05 was considered statistically significant.

## RESULTS

### Literature Search and Eligible Studies

**FIGURE 1:**
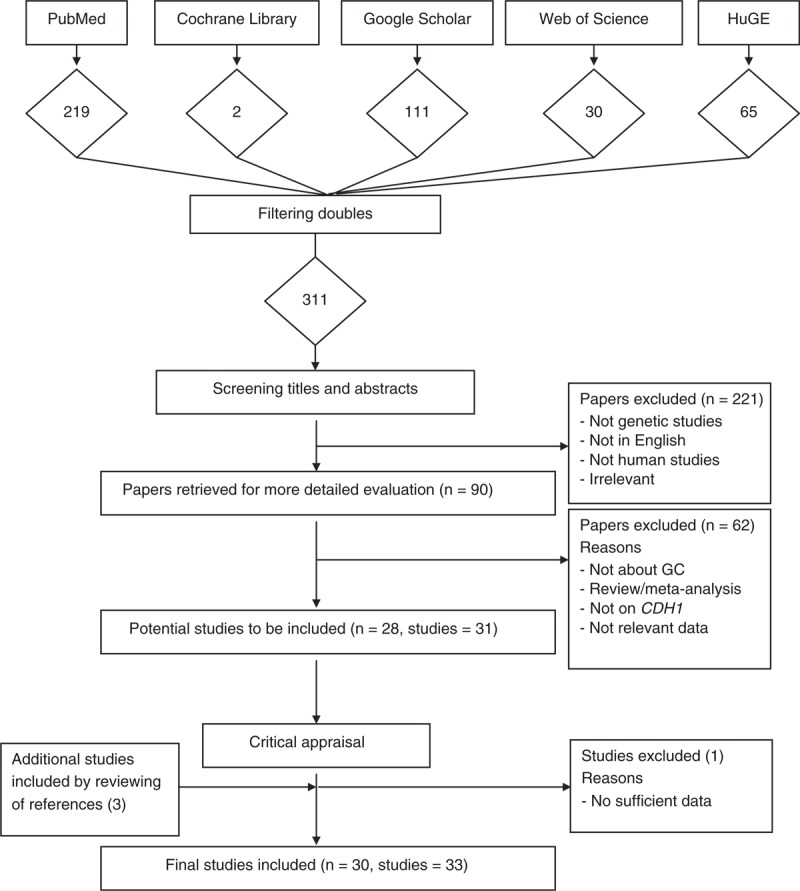
Flow diagram of the selection process of the studies included in the meta-analyses. Please see the Methods section for additional details. *CDH1* = E-cadherin gene, GC = gastric cancer.

Figure 1 is the flow diagram showing the selection of studies to be included in our analysis. Using our predefined search strategy, we identified a total of 311 potential publications through our initial search. After screening the abstracts of these studies, 221 were excluded either because they were irrelevant, not about human subjects, not genetic studies, or not published in English. The remaining 90 studies were retrieved for more detailed evaluations, which excluded an additional 62 studies because they were irrelevant, there were not sufficient data, the outcome of interest was not GC, or they were meta-analyses or review studies. This left 28 potentially relevant publications (with 31 studies) to be included in our analysis. A further review of the references of these studies and review articles identified 3 more studies. Further exploration of the data from these studies excluded 1 more study because of insufficient data. A total of 33 studies from 30 published articles met the eligibility criteria and were included in our analyses.^21–57^

All qualified publications were published since 2002 and had sample sizes ranging from 14 to 1197 (Table 2). Prevalence of GC ranged from 16% to 88%. Of these 33 studies, 22 studies reported association results for C-160A, 4 studies for rs1801552, rs3743674, and rs5030625, and 3 studies for rs1801026. Two studies investigated the association of GC with rs2010724, 2296-616G>C, and rs33964119. The combined study population included 9593 participants in the meta-analysis of C-160A, 1563 of rs1801552, 1993 of rs3743674, 2048 of rs5030625, 1373 of rs1801026, 783 of rs2010724, 771 of 2296-616G>C, and 447 of rs33964119. In addition to the 8 genetic variants included in the respective meta-analyses, the association between GC and 17 additional genetic variants in *CDH1* was reported in individual studies (or calculated based on individual studies). These results, together with results obtained from our mea-analyses, were included in our gene-based analysis. Moreover, the association of the promoter hypermethylation of *CDH1* with GC has been examined in 8 studies, and meta-analysis was performed to explore the effect of promoter hypermethylation on GC risk. DNA methylation was measured similarly across studies (ie, bisulfate treatment followed by methylation-specific polymerase chain reaction).

**TABLE 2:**
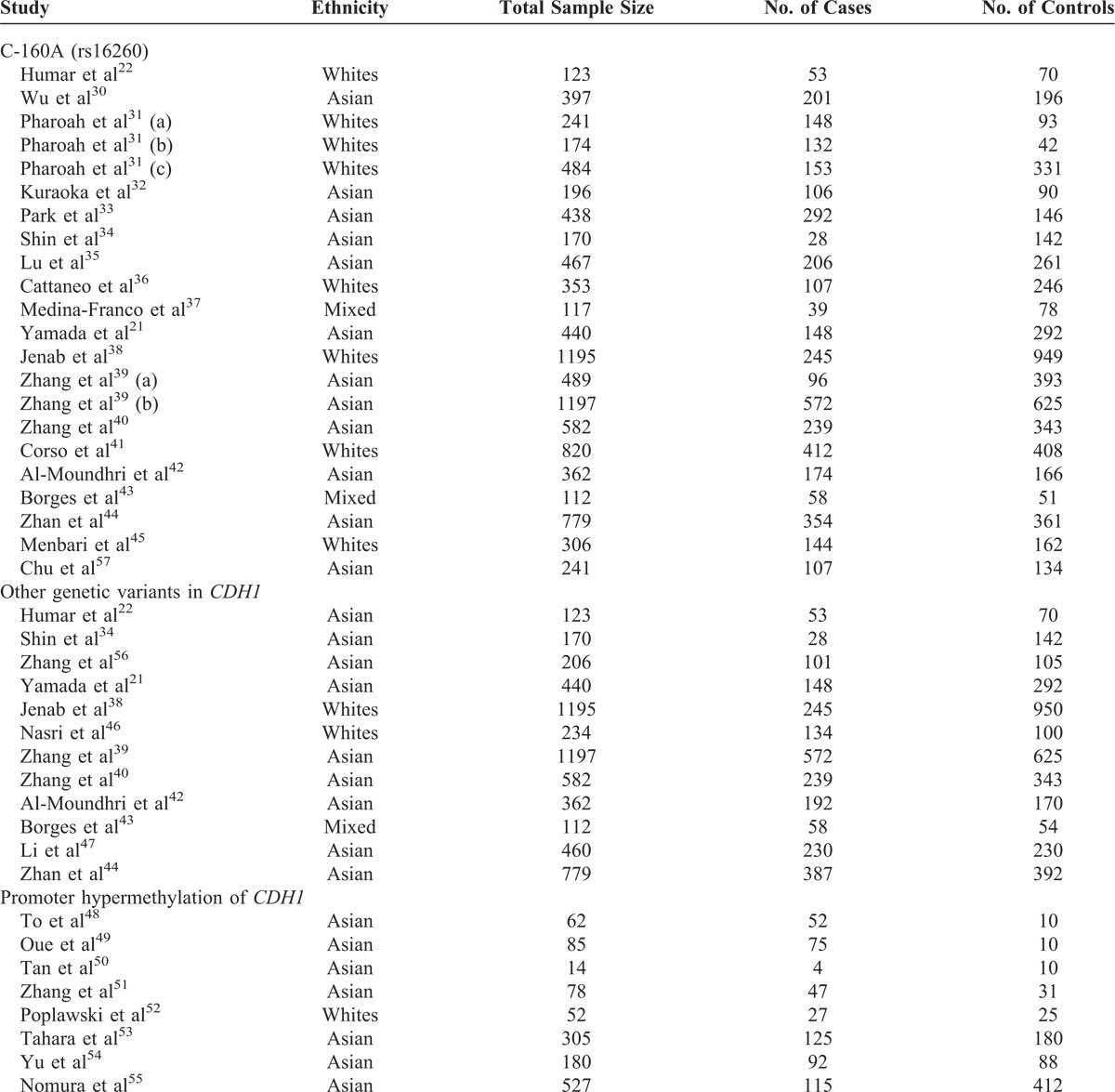
Characteristics of Studies Included in the Meta-Analyses

### Assessment of Publication Bias

**FIGURE 2:**
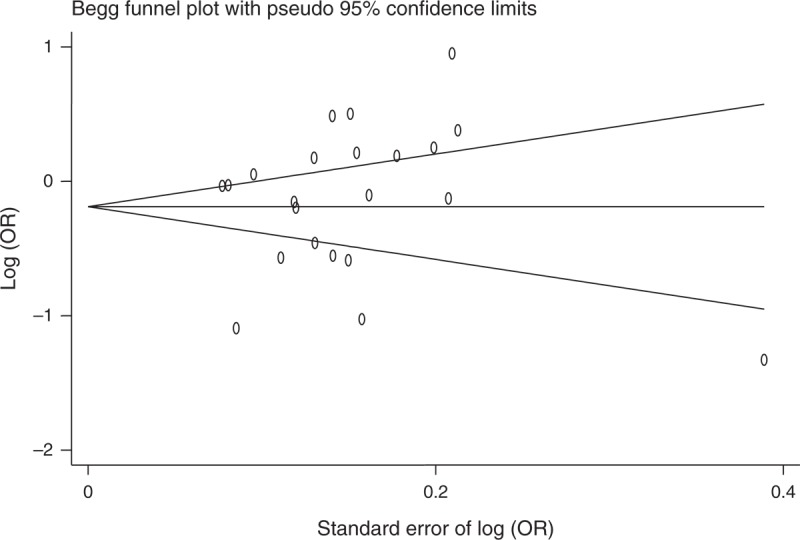
Funnel plot for meta-analysis of C-160A in *CDH1*. The x-axis is the standard error of the log-transformed OR (log [OR]), and the y-axis is the log (OR). The horizontal line in the figure represents the overall estimated log (OR). The 2 diagonal lines represent the pseudo 95% confidence limits of the effect estimate. *CDH1* = E-cadherin gene, log (OR) = log-transformed OR, OR = odds ratio.

**FIGURE 3:**
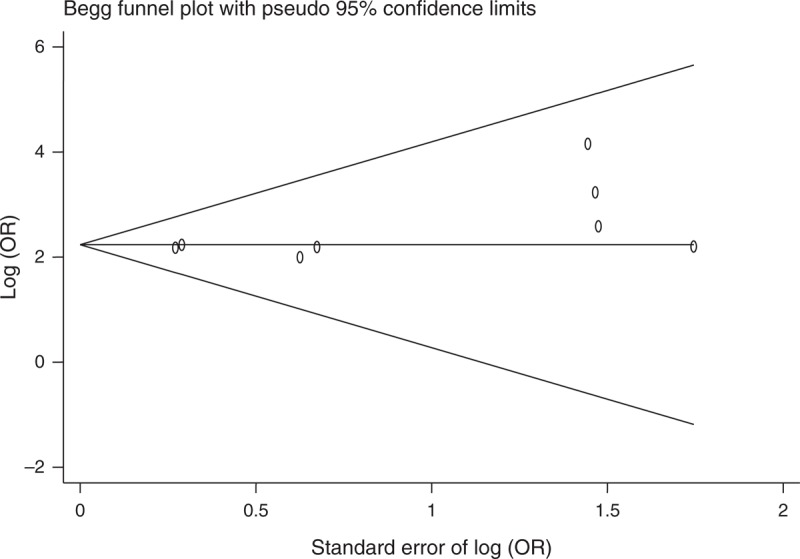
Funnel plot for meta-analysis of promoter hypermethylation of *CDH1*. The x-axis is the standard error of the log-transformed OR (log [OR]), and the y-axis is the log (OR). The horizontal line in the figure represents the overall estimated log (OR). The 2 diagonal lines represent the pseudo 95% confidence limits of the effect estimate. *CDH1* = E-cadherin gene, log (OR) = log-transformed OR, OR = odds ratio.

Both funnel plot and Egger test were used to assess publication bias. There was no evidence of publication bias for the meta-analysis of C-160A (*P* = 0.380, Figure 2). We found no evidence of publication bias for the meta-analysis of rs1801552, rs3743674, rs5030625, rs1801026 (all *P*s >0.38). There was some evidence of publication bias for the meta-analysis for promoter hypermethylation of *CDH1* (*P* = 0.128, Figure 3). Assessment of publication bias for the meta-analysis of other SNPs is not meaningful due to the low number of studies included in the corresponding meta-analysis.

### Association of C-160A With GC

We calculated the association between the C-160A in *CDH1* and GC assuming 4 different genetic models (additive, recessive, dominant, and allelic). Due to space limitations, we only present the results using an additive model. Results obtained using other models can be found in the supplementary materials.

**FIGURE 4:**
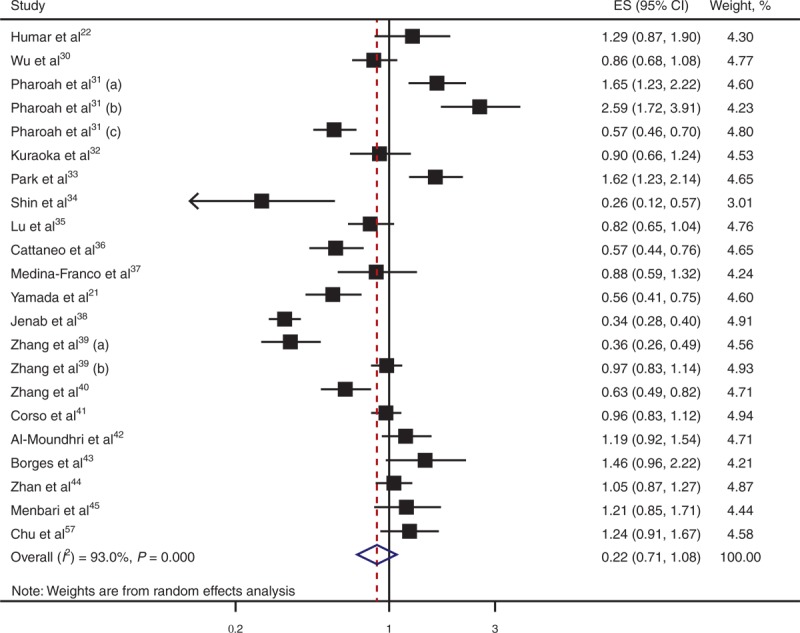
Forest plot for meta-analysis of C-160A in *CDH1*. Each study is represented by a square, whose area is proportional to the weight of the study. The overall effect from meta-analysis is represented by a diamond whose width represents the 95% CI for the estimated OR. *CDH1* = E-cadherin gene, CI = confidence interval, ES = effect size, OR = odds ratio.

Of the 22 studies included in our meta-analysis, 10 showed significant association between C-160A and GC (Table 3). Specifically, 3 studies^31,33^ indicated that compared with CC carriers, those carrying each additional copy of the A allele had increased risk of GC, whereas the other 7 studies reported decreased risk. Our meta-analysis indicates no significant association of C-160A with GC (OR = 0.88; 95% CI, 0.71–1.08; *P* = 0.215; Table 3, Figure 4). We found no significant association using different genetic models (Figures S1–S3 [http://links.lww.com/MD/A44, http://links.lww.com/MD/A45, and http://links.lww.com/MD/A46], Forest plots for meta-analysis of C-160A in *CDH1*; Tables S1–S3 [http://links.lww.com/MD/A47, http://links.lww.com/MD/A48, and http://links.lww.com/MD/A49], Meta-analysis of the association of C-160A in *CDH1* with GC).

**TABLE 3:**
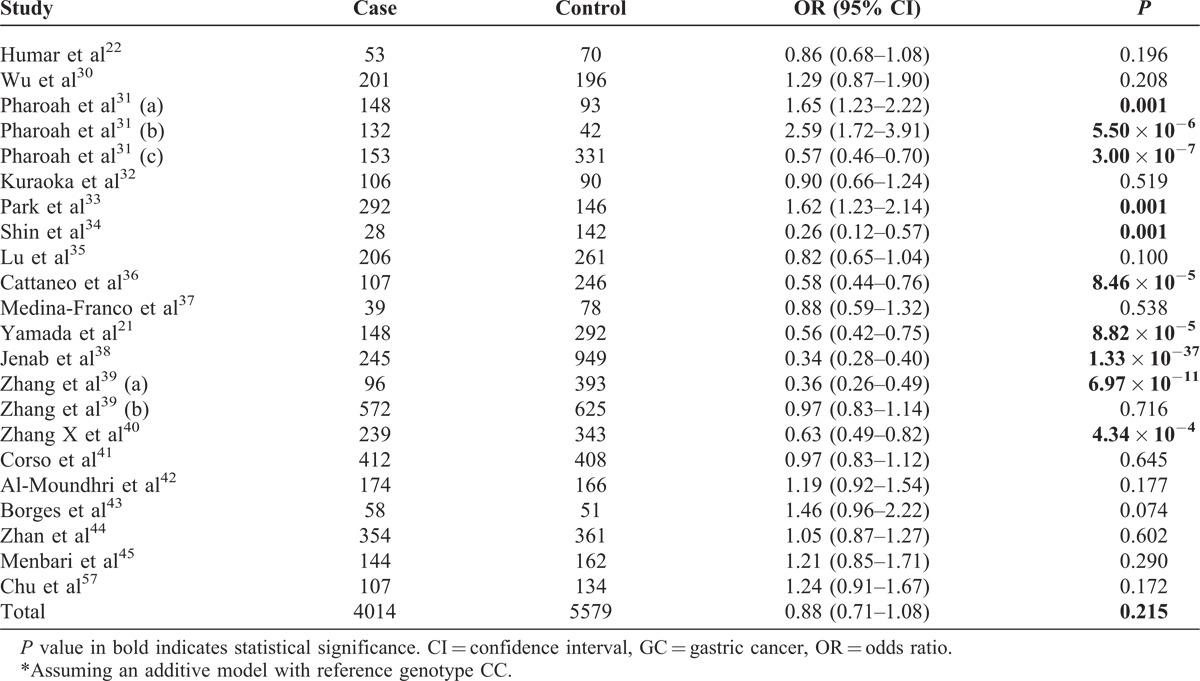
Meta-Analysis of the Association of GC and C-160A in CDH1*

### Association of C-160A With Subtypes of GC

We found significant association of C-160A with cardia (OR = 0.21; 95% CI, 0.11–0.41; 2.60 × 10^−6^), intestinal (OR = 0.66; 95% CI, 0.49–0.90; *P* = 0.008), and diffuse GCs (OR = 0.57; 95% CI, 0.40–0.82; *P* = 0.002). The association of C-160A with noncardia GC is of bottom line significance (OR = 0.65; 95% CI, 0.42–1.01; *P* = 0.054; Table 4).

**TABLE 4:**
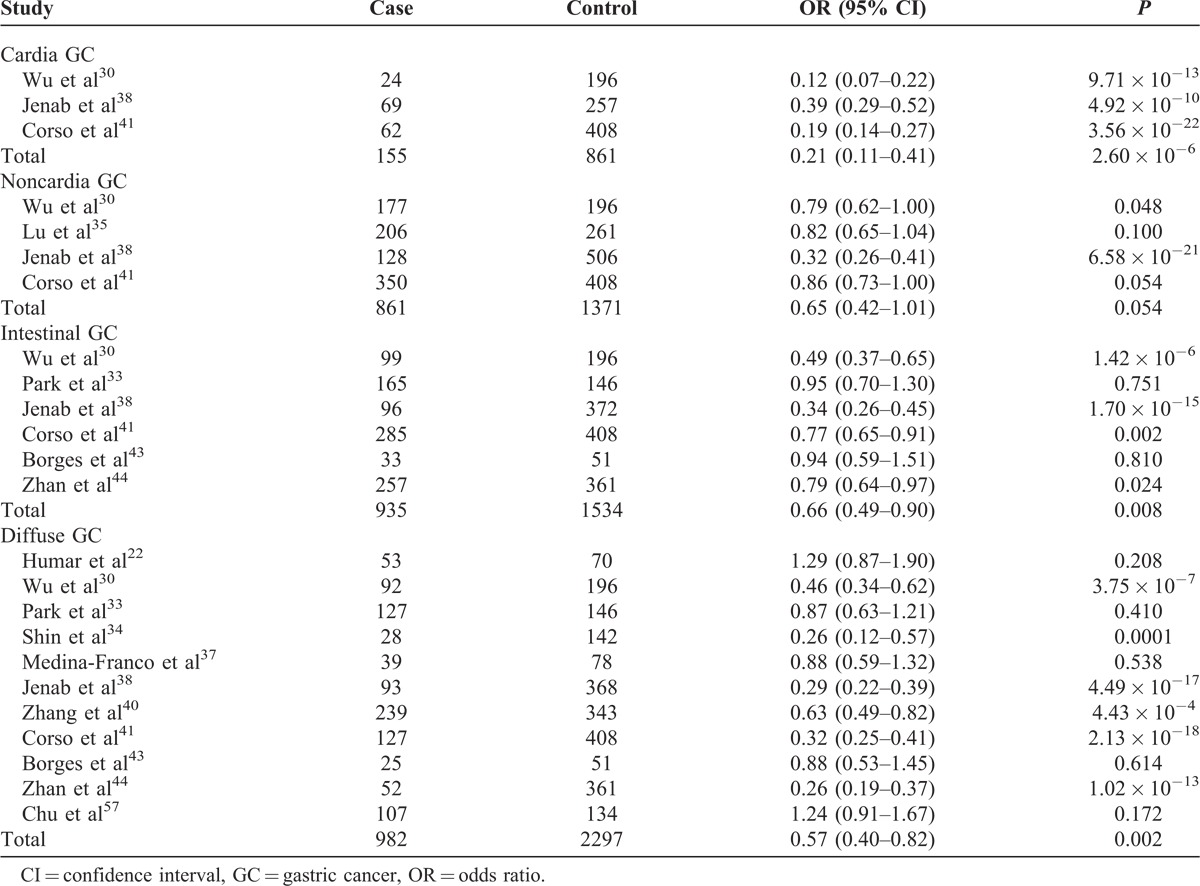
Association of C-160A With Subtypes of GC

### Association of Other Genetic Variants With GC

Our meta-analysis of the less-studied genetic variants in *CDH1* found no significant association with GC (Table 5). However, several genetic variants that appeared in single studies showed significant association with GC. Specifically, 7 genetic variants from a single study^38^ showed strong association with GC, whereas 1 other genetic variant (rs1125557) from another individual study^46^ also exhibited significant association with GC (*P* = 7.53 × 10^−5^).

**TABLE 5:**
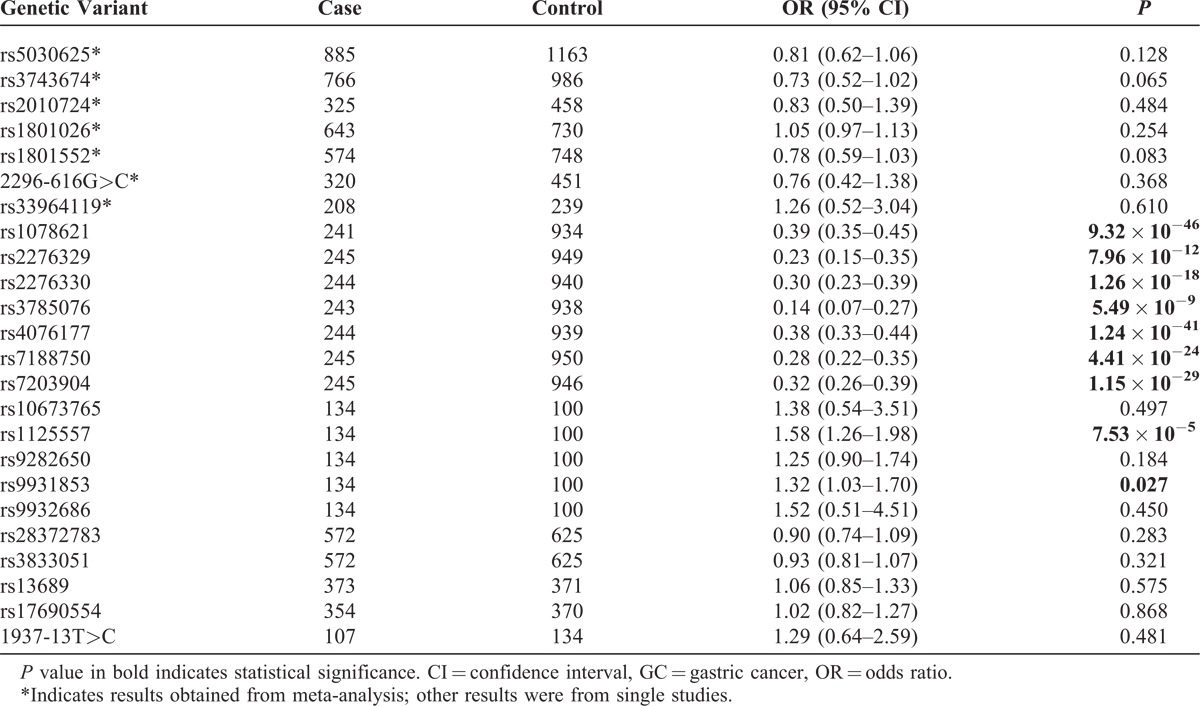
Association of Other Genetic Variants in *CDH1* With GC

### Gene-Based Analysis

To examine the cumulative association of multiple genetic variants in *CDH1* with GC, we performed a gene-based analysis using all the *P* values we obtained for each individual genetic variant in *CDH1*. Additionally, we examined whether the association varies in meta-studies only (including only results for genetic variants covered in meta-analyses) and in individual studies only (including only results for genetic variants that appeared in single studies). Our gene-based analysis indicated a significant association between the genetic variants in *CDH1* and GC (all *P*s <10^−5^). The association held when pooling results from only individual-studies, but disappeared when only results from meta-studies were included, indicating that the observed gene-based association was driven mainly by results from the less-studied genetic variants (Table S4 [http://links.lww.com/MD/A50], Gene-based analysis with GC). We would like to caution against over interpretation of the results from individual studies because, due to inadequate number of studies, it is not possible to determine whether there is selective reporting that can lead to inflation of the *P* values.

### Association of Promoter Hypermethylation of *CDH1* With GC

**FIGURE 5:**
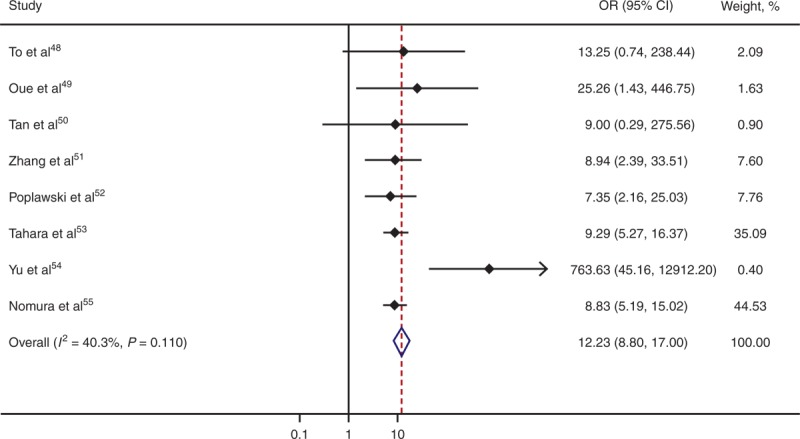
Forest plot for meta-analysis of promoter hypermethylation of *CDH1*. Each study is represented by a square whose area is proportional to the weight of the study. The overall effect from meta-analysis is represented by a diamond whose width represents the 95% CI for the estimated OR. *CDH1* = E-cadherin gene, CI = confidence interval, OR = odds ratio.

Our meta-analysis of 8 studies showed very strong and significant association of promoter hypermethylation of *CDH1* with GC (OR = 12.23; 95% CI, 8.80–17.00; *P* = 1.42 × 10^−50^; Table 6, Figure 5). More specifically, of the 8 studies, 1 study^50^ showed no association of promoter hypermethylation of *CDH1* with GC, probably due to insufficient statistical power resulting from limited sample size (n = 14), and another study^48^ showed marginal association (*P* = 0.080). All the other 6 studies indicated that promoter hypermethylation of *CDH1* is significantly associated with increased risk of GC.

**TABLE 6:**
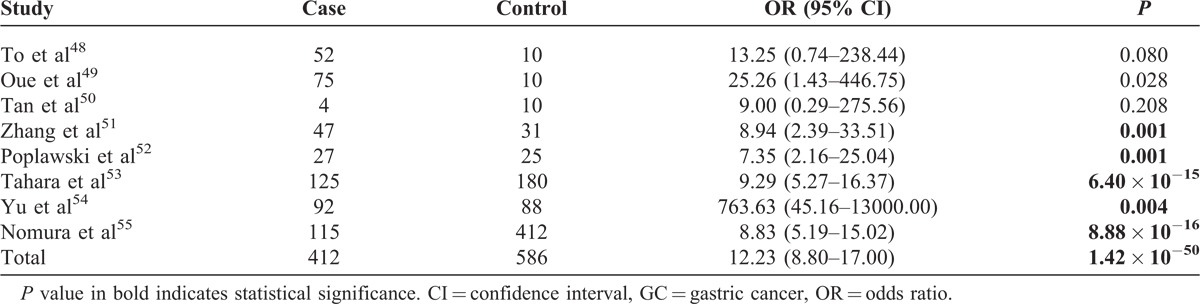
Association of Promoter Hypermethylation of *CDH1* With GC

### Sensitivity Analysis

There were 4 studies in which the genotype in the control group did not meet HWE.^32,40,44,45^ After excluding these 4 studies, our meta-analysis again indicated no significant association of C-160A with GC (OR = 0.87; 95% CI, 0.68–1.11; *P* = 0.261). There were 12 studies from 11 articles and 8 studies from 6 articles examining the association of C-160A with GC in Asian and white participants, respectively. Our meta-analysis based on these studies found no significant association of C-160A with GC in Asian (OR = 0.82; 95% CI, 0.66–1.02; *P* = 0.075) and white participants (OR = 0.94; 95% CI, 0.60–1.47; *P* = 0.793; Table S5 [http://links.lww.com/MD/A51], Meta-analysis of C-160A by ethnic groups). These results do not change after excluding studies that did not meet HWE.

There were 1 study^30^ and 2 studies^38,41^ examining the association of C-160A with cardia GC in Asian and white participants, respectively. A significant association was found between C-160A and GC in both Asian (OR = 0.12; 95% CI, 0.07–0.22; *P* = 9.71 × 10^−13^) and white participants (OR = 0.27; 95% CI, 0.14–0.55; *P* = 2.50 × 10^−4^; Table S6 [http://links.lww.com/MD/A52], Association with cardia and noncardia GC by ethnic groups). There were 2 studies examining the association of C-160A with noncardia GC in Asian^30,35^ and white participants.^38,42^ Our meta-analysis found significant association in Asian (OR = 0.80; 95% CI, 0.68–0.95; *P* = 0.011) but not in white participants (OR = 0.53; 95% CI, 0.20–1.37; *P* = 0.190). There were 3 studies^30,33,44^ and 2 studies^38,41^ examining the association of C-160A with intestinal GC in Asian and white participants, respectively. Our meta-analysis based on these studies found no significant association in Asian (OR = 0.72; 95% CI, 0.50–1.02; *P* = 0.066) and white participants (OR = 0.52; 95% CI, 0.23–1.14; *P* = 0.103; Table S7 [http://links.lww.com/MD/A53], Association with intestinal and diffuse GC by ethnic groups). There were 6 studies and 3 studies examining the association of C-160A with diffuse GC in Asian and white participants, respectively. Our meta-analysis based on these studies indicated significant association of C-160A with diffuse GC in Asian participants (OR = 0.55; 95% CI, 0.35–0.87; *P* = 0.011) but not in white participants (OR = 0.49; 95% CI, 0.22–1.09; *P* = 0.081; Table S7 [http://links.lww.com/MD/A53], Association with intestinal and diffuse GC by ethnic groups). However, after excluding the studies in which genotype in the control group did not meet HWE (n = 2), the association in Asian participants is no longer statistically significant (OR = 0.64; 95% CI, 0.36–1.13; *P* = 0.126).

## DISCUSSION

In this study, we conducted an extensive literature search for publications on the association of GC with genetic variants in and promoter hypermethylation of *CDH1*. We provided an updated meta-analysis on the widely studied genetic variant C-160A. Our analysis showed that C-160A is not associated with GC, either in the overall population, or in Asian or white participants. However, within a very limited set of articles that evaluated subtypes of GC, we found significant association of C-160A with cardiac, intestinal, and diffuse GC. We found that the promoter hypermethylation of *CDH1* is strongly associated with GC, indicating potential epigenetic influences in the carcinogenesis and development of GC. To the best of our knowledge, this is the most comprehensive meta-analysis on the association of GC with a number of genetic variants in *CDH1*, and with promoter methylation of *CDH1*.

In the meta-analysis of C-160A, we identified significant heterogeneity between the studies included for analysis (*I*^2^ = 93.0%; 95% CI, 90.6%–94.7%). Identifying the source of heterogeneity is challenging with limited information provided in many studies. Variation in patient characteristics might be an important source of heterogeneity. Some studies used matched controls (eg, age and sex matched),^21,22,35,37,38,43,46^ whereas most other studies did not perform matching. Other patient characteristics, such as smoking behavior, *H pylori* infection, and tumor location, can also contribute to the heterogeneity of the included studies in the meta-analyses.

Of the 24 less-studied genetic variants in *CDH1*, our analysis found multiple genetic variants showing significant association with GC. Specifically, 1 study by Jenab et al^38^ reported findings for 7 less-studied SNPs and all of them showed significant association with GC. Another study^46^ showed that rs1125557 was significantly associated with GC (Table 5). The gene-based analysis indicated that these less-studied genetic variants other than C-160A cumulatively confer significant genetic susceptibility of GC (Table S4 [http://links.lww.com/MD/A50], Gene-based analysis with GC). Realizing that the observed gene-based association might be driven by the results reported in the study by Jenab et al,^38^ in sensitivity analysis we dropped that study from the gene-based analysis and still observed significant gene-based association (all *P*s <0.003). The SNP rs1125557 is in high linkage disequilibrium (LD) with C-160A (D′ = 1, SNP annotation and proxy search, http://www.broadinstitute.org/mpg/snap/ldsearchpw.php). Given the high LD, we feel that the significant finding was probably because of the small sample size based on which the result was reported.^46^ Studies on functional outcomes of these less-studied genetic variants in *CDH1* are scarce, and further studies are needed to elucidate whether and how they function in influencing disease susceptibility.

DNA methylation is the most extensively studied epigenetic modification, and plays an important role in regulating gene expression and cell differentiation. Aberrant DNA methylation leads to silencing of tumor suppressor genes or loss of oncogene repression, and therefore is an important mechanism in the initiation and development of GC.^58^ The precise molecular mechanism underlying the association of promoter hypermethylation of *CDH1* with GC remains to be understood. A key challenge remains whether changes in methylation are a cause or an effect of the pathological process. Although some studies suggest that altered methylation in *CDH1* might be involved in carcinogenesis of GC but not development of GC,^55^ others indicate that accumulation of aberrant methylation might be an important mechanism for GC development.^48^ There are also studies indicating that the accumulation of DNA methylation might be caused by proliferative changes during tumor progression.^49^ Moreover, *CDH1* methylation seems to be age related,^51^ making it more complicated to disentangle the exact role of methylation in the initiation and development of GC. More future large-scale studies are needed that examine subjects at risk of developing GC as well as subjects with GC to better elucidate whether and how *CDH1* promoter hypermethylation is implicated in GC initiation and development.

Our study has some limitations. Since relevant data were not available, our meta-analysis could not adjust for confounding factors such as age, sex, smoking behavior, or *H pylori* infection. First, future studies are needed to validate our results—especially large consortium studies that provide control for such confounding factors. Second, some meta-analyses were based on few studies, and the gene-based analysis used some results from individual studies. Third, sensitivity analyses by ethnicity are limited because race information was not available in all studies. Fourth, due to the limited number of studies included in some of the meta-analyses, we could not test publication bias for them. This might lead to bias in the resulting data, and subsequently influence the validity of the gene-based analysis. Finally, there are other types of genetic variations that are not included in our study, such as copy number variation that was recently reported to be associated with GC.^59,60^

In summary, in this study, we performed meta-analyses to analyze the genetic and epigenetic effect of *CDH1* on GC risk. We found no significant association of the widely studied genetic variant C-160A with GC. However, a limited number of studies suggest that C-160A may be associated with subtypes of GC in different ethnic groups, and we identified some other genetic variants showing significant association with GC. Gene-based analysis indicated that the previously studied variants cumulatively influence GC susceptibility. Meta-analysis on the promoter hypermethylation of *CDH1* suggests that epigenetics also plays a critical role in the carcinogenesis of GC. Future studies with large sample sizes that control confounding risk factors and/or intensively interrogate CpG sites in *CDH1* are needed to validate the results found in this study and explore additional epigenetic loci that affect GC risk.
